# Technology-assisted training of arm-hand skills in stroke: concepts on reacquisition of motor control and therapist guidelines for rehabilitation technology design

**DOI:** 10.1186/1743-0003-6-1

**Published:** 2009-01-20

**Authors:** Annick AA Timmermans, Henk AM Seelen, Richard D Willmann, Herman Kingma

**Affiliations:** 1 Faculty of Biomedical Technology, Technical University Eindhoven, Den Dolech 2, 5600 MB Eindhoven, the Netherlands; 2 Rehabilitation Foundation Limburg (SRL), Research Dept, Zandbergsweg 111, 6432 CC Hoensbroek, the Netherlands; 3 Philips Research Europe, Dept Medical Signal Processing, Weisshausstrasse 2, 52066 Aachen, Germany; 4 Department of ORL-HNS, Maastricht University Medical Center, PO Box 5800, 6202 AZ Maastricht, the Netherlands

## Abstract

**Background:**

It is the purpose of this article to identify and review criteria that rehabilitation technology should meet in order to offer arm-hand training to stroke patients, based on recent principles of motor learning.

**Methods:**

A literature search was conducted in PubMed, MEDLINE, CINAHL, and EMBASE (1997–2007).

**Results:**

One hundred and eighty seven scientific papers/book references were identified as being relevant. Rehabilitation approaches for upper limb training after stroke show to have shifted in the last decade from being analytical towards being focussed on environmentally contextual skill training (task-oriented training). Training programmes for enhancing motor skills use patient and goal-tailored exercise schedules and individual feedback on exercise performance. Therapist criteria for upper limb rehabilitation technology are suggested which are used to evaluate the strengths and weaknesses of a number of current technological systems.

**Conclusion:**

This review shows that technology for supporting upper limb training after stroke needs to align with the evolution in rehabilitation training approaches of the last decade. A major challenge for related technological developments is to provide engaging patient-tailored task oriented arm-hand training in natural environments with patient-tailored feedback to support (re) learning of motor skills.

## Background

**Figure 1:**
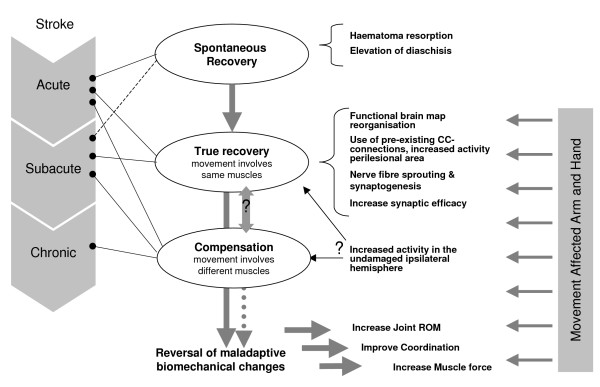
**Declarative model of motor recovery after stroke**. (CC = corticortical).

Stroke is the third leading cause of death in the USA and may cause serious long-term disabilities for its survivors [1]. The World Health Organisation (WHO) estimates that stroke events in EU countries are likely to increase by 30% between 2000 and 2025 [2]. Stroke patients may be classified as being in an acute, subacute or chronic stage after stroke. Although several restorative processes can occur together in different stages after stroke (figure 1), it can be said that spontaneous recovery through restitution of the ischemic penumbra and resolution of diaschisis takes place more in the acute stage after stroke (especially in the first four weeks [3]). Repair through reorganisation, supporting true recovery or, alternatively, compensation, may also take place in the subacute and chronic phase after stroke [3]. In true recovery, the same muscles as before the injury are recruited through functional reorganisation in the undamaged motor cortex or through recruitment of undamaged redundant cortico-cortical connections [4]. In compensation strategies, alternative muscle coalitions are used for skill performance. To date, central nervous system adaptations behind compensation strategies have not been clarified. In any case, learning is a necessary condition for true recovery as well as for compensation [3] and can be stimulated and shaped by rehabilitation; and this most, but not solely, in the first 6 months after the stroke event [5]. However, little is currently known about how different therapy modalities and therapy designs can influence brain reorganisation to support true recovery or compensation.

Persons who suffer from functional impairment after stroke often have not reached their full potential for recovery when they are discharged from hospital, where they receive initial rehabilitation [6-8]. This is especially the case for the recovery of arm-hand function, which lags behind recovery of other functions [9]. A major obstacle for rehabilitation after hospital discharge is geographical distance between patients and therapists as well as limited availability of personnel [10]. This leads to high levels of patient dissatisfaction for not receiving adequate and sufficient training possibilities after discharge from hospital [11]. Four years after stroke, only 6% of stroke patients are satisfied with the functionality of their impaired arm [8].

As therapy demand is expected to increase in future, an important role emerges for technology that will allow patients to perform training with minimal therapist time consumption [12-14]. With such technology patients can train much more often, which leads to better results and faster progress in motor (re) learning [15]. There is scientific evidence that guided home rehabilitation prevents patients from deteriorating in their ability to undertake activities of daily living [16,17], may lead to functional improvement [6,16,18-20], higher social participation and lower rates of depression [20].

This setting has motivated multidisciplinary efforts for the development of rehabilitation robotics, virtual reality applications, monitoring of movement/force application and telerehabilitation.

The aim of this paper is 1) to bring together a list of criteria for the development of optimal upper limb rehabilitation technology that is derived from the fields of rehabilitation and motor control and 2) to review literature as to what extent current technological applications have followed the evolution in rehabilitation approaches in the last decade. While a wealth of technologies is currently under development and shows a lot of promise, it is not the aim of this article to give an inventory of technology described in engineering databases. For an overview of such work, readers are referred to Riener et al. [21]. As this article is written from a therapy perspective, only technology that has been tested through clinical trial(s) will be evaluated.

This information may guide persons that are active in the domain of rehabilitation technology development in the conceptualisation and design of technology-based training systems.

## Methods

A literature search was conducted using the following databases: PubMed, MEDLINE, CINAHL, and EMBASE. The database search is chosen to be clinically oriented, as it is the authors aim to 1) gather guidelines for technology design from the fields of motor learning/rehabilitation and 2) to evaluate technology that has been tested through clinical trial(s).

Papers published in 1997–2007 were reviewed. The following MeSH keywords were used in several combinations: "Cerebrovascular Accident" not "Cerebral Palsy", "Exercise Therapy", "Rehabilitation", "Physical Therapy" not "Electric Stimulation Therapy", "Occupational Therapy", "Movement", "Upper Extremity", "Exercise", "Motor Skills" or "Motor Skill Disorders", "Biomedical Technology" or "Technology", "Automation", "Feedback", "Knowledge of results", "Tele-rehabilitation" as well as spelling variations of these terms. Additionally, information from relevant references cited in the articles selected was used. After evaluation of the content relevance of the articles that resulted from the search described above, 187 journal papers or book chapters were finally selected, forming the basis of this paper.

## Results

### State-of-the-art approaches in motor (re)learning in stroke and criteria for rehabilitation technology design

#### General

The International Classification of Functioning, Disability and Health (ICF) [22,23] classifies health and disease at three levels: 1) Function level (aimed at body structures and function), 2) Activity level (aimed at skills, task execution and activity completion) and 3) Participation level (focussed on how a person takes up his/her role in society). This classification has brought about awareness that addressing "health "goes further than merely addressing "function level", as has been the case in healthcare until the middle of the last decade.

Rehabilitation after stroke has evolved during the last 15 years from mostly analytical rehabilitation methods to also including task-oriented training approaches. Analytical methods address localised joint movements that are not linked to skills, but to function level. Task-oriented approaches involve training of skills and activities aimed at increasing subject's participation. Since Butefisch et al [24] started challenging conventional physiotherapy approaches that focus on spasticity reduction, a new focus on addressing paresis and disordered motor control has emerged [25-28]. Several authors advocate the use rehabilitation methods that include repetition of meaningful and engaging movements in order to induce changes in the cerebral cortex that support motor recovery (brain plasticity) [29-32]. Knowing that training effects are task-specific [33] and that to obtain improvement in "health" an improvement on different levels of functioning is required [22], it is now generally accepted that sensory-motor training is a total package, consisting of several stages: a) training of basic functions (e.g. muscle force, range of motion, tonus, coordination) prerequisite to skill training, b) skill training (cognitive, associative and autonomous phase) and c) improvement of endurance on muscular and/or cardiovascular level [34]. Apart from active therapy approaches where a patient consciously participates in a motor activity, also recent views on therapy goal setting, motivation aspects of therapy and feedback delivery on exercise performance are discussed and used for setting therapist criteria for rehabilitation technology (for an overview see table 1). Where possible, the authors aim to link training methods to neurophysiologic recovery processes.

**Table 1:** Checklist of criteria/guidelines for robotic and sensor rehabilitation technology, based on motor learning principles

**Criteria related to therapy approaches**
- Training should address function, activity and participation levels by offering strength training, task-oriented/CIMT training, bilateral training.
- Training should happen in the natural environmental context.
- Frequent movement repetition should be included.
- Training load should be patient and goal-tailored (differentiating strength, endurance, co-ordination).
- Exercise variability should be on offer.
- Distributed and random practise should be included.

**Criteria related to motivational aspects**

- Training should include fun & gaming, should be engaging
- The active role of the patient in rehabilitation should be stimulated by:
○ therapist independence on system use.
○ individual goal setting that is guided to be realistic.
○ self-control on delivery time of exercise instructions and by feedback that is guided to support motor learning.
○ control in training protocol: exercise, exercise material, etc.

**Criteria related to feedback on exercise performance**

- KR (average & summary feedback) and KP should be available (objective standardized assessment of exercise performance is necessity).
- Progress Components:
○ fading frequency schedule (from short to long summary/average lengths)
○ from prescriptive to descriptive feedback
○ from general (e.g. sequencing right components) to more specific feedback (range of movement, force application, etc)
○ from simple to more complex feedback (according to cognitive level).
- Empty time slot for performance evaluation before and after giving feedback.
- Guided self-control on timing delivery feedback.
- Feedback on error and correct performance.

#### Active therapy approaches

To determine the evidence for physical therapy interventions aimed at improving functional outcome after stroke, Van Peppen et al. [27] conducted a systematic literature review including one hundred twenty three randomised controlled clinical trials and 28 controlled clinical trials. They found that treatment focussing only on function level, as does muscle strengthening and/or nerve stimulation, has significant effects on function level but fails to influence the activity level. So, even if e.g. strength is an essential basis for good skill performance [35], more aspects involved in efficient movement strategies need to be addressed in order to train optimal motor control. Active training approaches, with most evidence of impact on functional outcome after stroke are: task-oriented training, constrained induced movement therapy and bilateral arm training [27].

*Task-oriented training *stands for a repetitive training of functional (= skill-related) tasks. Task-oriented training has been clinically tested mostly for training locomotion [34,36-38] and balance [39]. It is, however, also known to positively affect arm-hand function recovery, motor control and strength in stroke patients [9,27,40-46]. The value of task-oriented training is seen in the fact that movement is defined by its environmental context. Patients learn by solving problems that are task-specific, such as anticipatory locomotor adjustments, cognitive processing, and finding efficient goal-oriented movement strategies. Efficient movement strategies are motor strategies used by an individual to master redundant degrees of freedom of his/her voluntary movement so that movement occurs in a way that is as economic as possible for the human body, given the fact that the activity result needs to be achieved to the best of the patient's ability. Training effects are task specific, with reduced effects in untrained tasks that are similar [3,33,47,48]. At the same time, impairments that hinder functional movement are resolved or reduced. All of these aspects contribute to more efficient movement strategies for skill performance [7,26,34,48,49].

Task-oriented training approaches are consistent with the ICF [22,50] as function level is addressed, as well as activity and participation level. Task-oriented training is proven to result in a faster and better treatment outcome than traditional methods, like Bobath therapy, in the acute phase after stroke [51]. Without further therapy input however, this differential effect is not maintained, suggesting that training needs to continue beyond the acute phase in order for its positive effect not to deteriorate [52]. *Constrained Induced Movement Therapy (CIMT) *is a specialised task-oriented training approach that has proven to improve arm hand function for stroke patients through several randomised clinical trials involving a large amount of patients [53-61]. The effects of CIMT training have found to persist even 1–2 years after the training was stopped [57]. CIMT comprises several treatment components such as functional training of the affected arm with gradually increasing difficulty levels, immobilisation of the patient's non-affected arm for 90% of waking hours and a focus on the use of the more affected arm in different everyday life activities, guided by shaping [56,62]. Shaping consists of consistent reward of performance, making use of the possibility of operant conditioning [3], which is an implicit or non-declarative learning process through association [63]. A disadvantage of CIMT training is that it requires extensive therapist guidance as well as an intensive patient practise schedule, which present obstacles for its wider acceptance by patients and therapists [64]. Efforts are currently undertaken to further develop automation of CIMT (AutoCITE therapy) [56].

*Bilateral arm training *includes simultaneous active movement of the paretic and the non-affected arm[65]. Bilateral arm training is a recent training method that has, through randomised clinical trials, proven to augment range of movement, grip strength and dexterity of the paretic arm [27,65-67].

It still is not fully understood which neurophysiological processes (fig. 1) support the positive clinical outcomes of rehabilitation approaches, not even in, e.g. CIMT, an approach extensively investigated [3,68]. Sensorimotor integration has been proven to be an important condition for motor learning [69]. Functional neuroimaging studies suggest that increased activity in the ipsilesional sensorimotor and primary motor cortex may play a role in the improvement of functional outcome after task-specific rehabilitation [68,70], such as task-oriented training [71,72] and CIMT [73,74]. Other study results suggest that motor recovery after CIMT training may occur because of a shift of balance in the motor cortical recruitment towards the undamaged hemisphere [68]. The latter rehabilitation-induced gains may be a progression in the cortical processes (e.g. by unmasking existing less active motor pathways) that support motor recovery in earlier phases after stroke [68]. Alternatively, increased ipsilateral motor cortex involvement may occur because of the subject engaging in more complex or precise movements. Ipsilateral motor cortex involvement may also facilitate compensation strategies for motor performance [68,70]. It is thought that patients who have substantial corticospinal tract damage are more likely to restore sensorimotor functionality by compensation through use of functionally related systems, whereas patients with partial damage are likely to recover through extension of residual areas [70]. Unfortunately, although it is well known that stroke patients may show true recovery as well as behavioural compensation [5], the phasing and interaction of both in any functional recovery process after stroke remains to be clarified. Outcome scales used in clinical rehabilitation trials do not allow the distinction between true recovery (same muscles as before lesion are involved in task performance) and compensation (different muscle coalitions are used for task performance) [3]. Future studies that combine electromyography and neuro-imaging of the central nervous system could shed light on these processes.

Regardless of the therapy approach used, the *training load *should be tailored to individual patient's capabilities and to treatment goals that are defined prior to training. Training goals can be, e.g. to increase muscle strength, endurance or co-ordination [75,76]. To obtain an improved muscle performance, training load needs to exceed the person's metabolic muscle capacity (overload principle) [77]. The training load for the patient is determined by the total time spent on therapeutic activity, the number of repetitions, the difficulty of the activity in terms of co-ordination, muscle activity type and resistance load, and the intensity, i.e. number of repetitions per time unit [78,79]. When, e.g. improvement of muscle strength is the goal of a set of exercises, the training load should be such that fatigue is induced after 6 to 12 exercise repetitions. This training load will be different for different patients and needs to be individually determined. When training muscle endurance or coordination is the goal, many repetitions are used (40–50 or more) against a submaximal load [79]. Distributed practice (a practice schedule with frequent rest periods) and random ordering of task-related exercises improves performance and learning [3,80]. A good interchange between loading and adequate rest intervals are necessary for the body to recuperate from acute effects of exercise such as muscle fatigue [79]. Also variability in exercises when training a certain task improves retention of learning effects [3].

Training schedules, although very much determinant for training effects, are too often determined on an empirical basis [78].

In line with rehabilitation, rehabilitation technologies should address all levels of the ICF classification. Upper limb skill training should, where possible, happen in an environment that is natural for the specific task that is trained, as motor skills are shown to improve more than when trained out of context [81,82]. Training programs on offer should support individual training goals by offering a personalized training load [77,79]. Also, the more differentiated and varied training programs can be offered to the patient, the better retention of learning effects and the higher the chance that a patient can and will choose the one that fits him/her best [3,35,49].

#### Personal Goal Setting

Active training approaches allow patients to take an active role in the rehabilitation process. This is especially stimulated when patients can exercise with some self-selected, well-defined and individually meaningful functional goals in mind (goal-directed approach). Personal goal setting encourages patient motivation, treatment adherence and self-regulation processes. It also provides a means for patient progress assessment (are goals attained and to which extent? – or not) and patient-tailored rehabilitation [83-86]. The tasks that are selected to work on, should be within the patient capabilities, so that self-efficacy and problem solving can be stimulated; even though exercising might be difficult initially [85,87].

A goal-directed approach includes several essential components: 1. selection of patient's goal from a choice that is guided to be "SMART" (= Specific, Measurable, Attainable, Realistic and Time specified), 2. analysis of patient's task performance regarding the selected goal, 3. both identification of the variables that limit patient's performance and identification of patient constraints as a basis of treatment strategy selection, 4. analysis of the intervention and patient's performance leads to structurally offered feedback that supports motor learning (described infra), 5. conscious involvement of the patient to learn from feedback via restoration of cognitive processes that are associated with functional movement and 6. finding strategies to determine individually which are the most effective solutions [85]. Goal attainment scaling (GAS) is an effective tool for the above described process and evaluation of training outcome. In GAS the patient defines a goal, as well as a range of possible outcomes for it on a scale from 0 (expected result) +/- 2. This implies that patient's progress is rated relative to the goal set at baseline [88,85]. For more information about goal setting and goal attainment scaling, the authors refer to Kiresuk et al [88].

It should be clear to the patient at every stage of the training which movements support which goals to avoid goal-confusion. To set up the exercise environment in a natural or realistic manner will support the latter [87].

It is important that also technology provides the opportunity for the patient to have an active role in his rehabilitation process through personal treatment goal setting.

#### Motivation, patient empowerment, gaming and support from friends/family

Overprotection of persons after stroke by family caregivers may lead to more depression and less motivation to engage in physical therapy programs [89]. But also overprotection by the therapist, undermines the active role a patient can have in his rehabilitation process [83,90]. Motor skill learning and retention of motor skills can be enhanced if a patient assumes control over practice conditions, e.g. timing of exercise instructions and feedback [91]. As reflection and attention are both important factors for explicit (declarative) motor learning [63], patients should be able to control that instructions and feedback are offered when they are able to learn from it. A balance has to be found between freedom and guidance to accommodate different stages of learning (cognitive, associative and autonomous stages of learning [92]). Bach-y-Rita et al. [93,94] supported, through literature review, the introduction of therapy for persons after stroke that is engaging and motivating in order to obtain patient alertness and full participation that optimises motor (re)learning. Improvement of arm-hand function in case-studies support the use of computer-assisted motivating rehabilitation as an inexpensive and engaging way to train [95] where joy of participation in the training should compensate its hardship [94,95]. As an increase in therapy time after stroke has been proven to favour ADL outcome [38], it is important that patients are motivated to comply. To stimulate exercise compliance, family support and social isolation are issues to be addressed [96].

#### Feedback

##### General

It is important that feedback of exercise performance is given based on motor control knowledge, as this enhances motor learning and positively influences motivation, self-efficacy and compliance [97-100]. Feedback on correct motor performance enhances motivation [80], while feedback on incorrect exercise performance is more effective in facilitating skill improvement [101,102].

**Figure 2:**
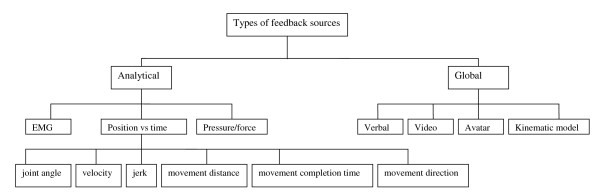
**Schematic presentation of types of augmented feedback sources for motor performance**.

Feedback from any skill performance is acquired through task-intrinsic feedback mechanisms and task-extrinsic feedback. Task-intrinsic feedback is provided through visual, tactile, proprioceptive and auditory cues to a person who performs the task. Task-extrinsic feedback or augmented feedback includes verbal encouragement, charts, tones, video camera material, computer generated kinematic characteristics (e.g. avatar) (fig 2).

Brain damage often impairs intrinsic feedback mechanisms of stroke patients, which means that they have to rely more on extrinsic feedback for motor learning. Although rather well understood for healthy subjects, information on the efficiency of augmented feedback in motor skill learning after stroke is scarce [100].

**Figure 3:**
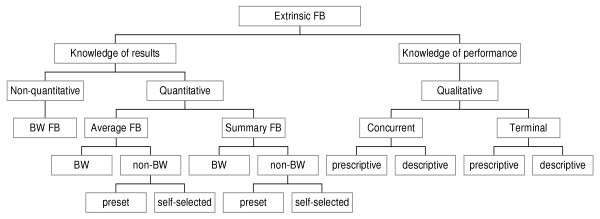
**Schematic presentation of extrinsic feedback components for motor performance**. (FB = feedback, BW = bandwidth).

Extrinsic feedback can be categorised as knowledge of results (KR) or knowledge of performance (KP), summary feedback (overview of results of previous trials) or average feedback (average of results of previous trials), bandwidth feedback, qualitative or quantitative feedback and can be given concurrently or at the end of task performance (terminal feedback) (fig 3) [34,100,103]. KR is externally presented information about outcome of skill performance or about goal achievement. KP is information about movement characteristics that led to the performance [80]. Both kinds of feedback are valuable [102,104,105], although there is some evidence that, for skill learning in general [106,107]and also specifically for persons after stroke [108], the use of KP during repetitive movement practice results in better motor outcomes. Van Dijk et al [109] performed a systematic literature search to assess effectiveness of augmented feedback (i.e. electromyographic biofeedback, kinetic feedback, kinematic feedback or knowledge of results). They found little evidence for differences in effectiveness amongst the different forms of augmented feedback.

#### Nature and timing of feedback addresses different stages of motor learning

Feedback needs to be tailored to the skill level of its receiver. Bandwidth feedback is a useful way of tailoring the feedback frequency to the individual patient, whereby the patients only receive a feedback signal when the amount of error is greater than a pre-set error range [80]. Beginners need simple information to help them approximate the required movement; more experienced persons need more specific information [100,110]. Novices seem to benefit more from prescriptive KP (stating the error and how to correct it), while for more advanced persons descriptive KP (stating the error) seems to suffice [80].

Two major systems in the brain, implicit and explicit learning/memory, can both contribute to motor learning [111]. Prescriptive feedback can make use of declarative or explicit learning processes, resulting in factual knowledge that can be consciously recalled from the long-term memory [34]. Vidoni et al [111] state that "explicit awareness of task characteristics may shape performance". Specific information may be offered as a sequence of 2 or more movement components (such as: keep your trunk stable against the back of your chair, then lower your shoulder girdle, then reach out for the cup, finally concentrate on grasping the cup). Declarative or explicit learning requires attention and awareness to enable information storage in the long-term memory, involving neural pathways from frontal brain areas, hippocampus and medial temporal lobe structures [34,111].

Descriptive feedback (e.g. "concentrate on movement selectivity") assumes that the patient has some experience with performing the movement and has learned by repetition how to correct through implicit or non-declarative learning strategies, such as associative learning (classical and operant conditioning) and/or procedural learning (skills and habits). Non-declarative learning occurs in the cerebellum (movement conditioning), the amygdala (involvement of emotion), and the lateral dorsal premotor areas (association of sensory input with movement). The information is stored in the long-term memory [63,34].

Choosing appropriate and patient-customised feedback is very complex and depends on the location and the type of the brain lesion [112,34]. Although frequently used by therapists, the use of declarative instructions/feedback for motor learning is questionable, especially when used in combination with non-declarative instructions/feedback [113,111]. Both learning mechanisms may compete for the use of memory processing capacity [111]. This may be the reason for the finding that feedback that is provided concurrently to movement (as in online feedback) has not been found to support motor learning as the learning effect does not persist after feedback is removed [114]. Also feedback that is given immediately after completion of movement may impede the use of intrinsic feedback for task performance analysis [115,100]. There is no experimental evidence for the optimal feedback delay after movement performance [80,34]. It has been shown that the KR delay should not be filled with other motor or cognitive skills that may interfere with learning of target movements [116,117]. Also the finding that subjective performance evaluation or estimation of specific characteristics of some of the movement-related components of a performed skill before and after KR/KP seem to benefit motor learning [118,115], is in support of these findings. Wulf [91] advocates allowing patients to choose the time of feedback delivery. This gives patients control, which can enhance motivation, potentially improving retention and transfer effects [91].

It seems more effective to give average or summary feedback than to give feedback after each trial [119,120] as the latter discourages variety in learning strategies (e.g. active problem solving-activities), leads to feedback dependency and possibly also to an attention-capacity overload [121]. The optimal number of trials summarised depends on the complexity of the task in relation to the performer's skill level [122]. Progressively reducing the feedback frequency (fading schedule strategy) might have a better retention of learning effects and better transfer effects, as the dependency of the performance on feedback decreases [34,100,120].

In summary, it can be stated that rehabilitation technology should provide both knowledge of results as well as knowledge of performance. A combination of error-based augmented feedback and feedback on correct movement characteristics of the performed movement is advisable to enhance learning and motivation. Active engagement of the patient in the feedback process is to be encouraged, by subjective performance evaluation and using the information for planning the next movement. Careful use of feedback that uses declarative learning is warranted.

### Technology supporting training of arm-hand function after stroke

For upper limb rehabilitation after stroke, two categories of rehabilitation systems will be described: robotic training systems and sensor-based training systems.

A wide variety of systems have been developed. Only those for which clinical data have been presented are discussed in this paper. These technologies may all be further enhanced using virtual reality techniques. However, it is not in the scope of this paper to discuss all virtual reality applications for stroke rehabilitation (for an overview see Sveistrup H. [123]). Thirty four studies, involving in total 755 patients, report testing by stroke patients of thirteen arm-hand-training systems. A short description is given for each of these systems. The number of clinical trials will be mentioned for each system, as well as the kind of trial and the total number of patients involved. More information (e.g. on amount of patients involved in each trial and outcome measures that were used) can be found in additional file 1 and table 2. For information about the quality aspects of the RCTs that are mentioned, the authors refer to a systematic review by Kwakkel et al [124].

**Table 2:** Overview of sensor technology used in stroke rehabilitation

**Name**	**Body area trained**	**Sensor-type**	**PA**	**FB**	**TDL**	**CT** **CCT** **RCT** **(n patients)**	**OCM**	**acute subacute chronic patients**
**Auto CITE (34)**	shoulder elbow forearm wrist hand	sensors built into workstation	CIMT	KR: number of successful repetitions	1	CCT (27)[56]	MAL, WMFT	chronic
				KP Encouragement		CT (7)[177]	MAL WMFT JHFT	chronic

(FB = feedback, PA = Physiotherapy Approach, CIMT = constrained induced movement therapy, TDL = therapist dependency level: 0 = no, 1 = minimal 2 = fully dependent, OCM = outcome measure, CT = clinical trial, CCT = controlled clinical trial, WMFT = Wolf Motor Function Test, MAL = Motor Activity Log).

#### Robotic training systems

Therapeutic robotics development started about 15 years ago at which time scientific evidence supporting rehabilitation approaches was much sparser. This has been a difficulty for development of technological rehabilitation systems in the past [125].

The upper limb robotic systems that exist until today can be classified roughly in passive systems (stabilising limb), active systems (actuators moving limb) and interactive systems [21]. Interactive systems are equipped with actuators as well as with impedance and control strategies to allow reacting on patient actions [21]. The interactive systems can be classified by the degrees of freedom (DOF) in which they allow movement to occur.

Existing interactive one-degree of freedom systems are e.g. Hesse's Bi-Manu-Track, Rolling Pin, Push & Pull [126,127], BATRAC [65] & the Cozens arm robot [128]. These systems are useful for stroke patients with lower functional levels (= proficiency level for skill related movement). Multi-degrees of freedom interactive robotic systems may be useful for patients with lower as well as higher functional levels.

One of the first robotic rehabilitation systems for upper limb training after stroke is **MIT-MANUS **developed by Krebs et al [12,129]. It allows for training wrist, elbow and shoulder movements by moving to targets, tracing figures and virtual reality task-oriented training. The robot allows two degrees of freedom. This enables training at patient function level, improving e.g. movement range and strength. The patient can train in passive, active and interactive (movement triggered or EMG-triggered) training modes. Patients with all levels of muscle strength can use the system. Visual, tactile and auditory feedback during movement is provided [12,125,130-134]. MIT-MANUS has been shown to improve motor function in the hemiparetic upper extremity of acute, subacute and chronic stroke patients in 5 clinical trials (CTs)[131,135-138] and 5 randomized clinical trials (RCTs) [139-143]. In total 372 persons were tested. This is close to half of the total number of stroke patients tested in technology-supported arm training trials until the end of 2007.

**MIME **(Mirror Image Movement Enhancer) [132,144-146] consists of a six degrees of freedom robot manipulator, which applies forces (assistance or resistance as needed) to a patient's hand through a handle that is connected to the end-effector of the robot. This robot treatment focuses on shoulder and elbow function. The MIME system can work in preprogrammed position and orientation trajectories. It can also be used in a configuration where the affected arm is to perform a mirror movement of the movement defined by the intact arm. The forearm can be positioned in a large range of positions and has therefore the possibility to let the patient exercise in complex movement patterns. Four modes of robot-assisted movement are available: passive, active-assisted, active-constrained and bimanual mode. The MIME system has been validated through 1 CT [147] and 3 RCTs [145,146,148], involving 76 chronic stroke patients.

**BI-MANU-TRACK **is a one degree of freedom system, designed by Hesse et al [126,127,149] to train forearm pro-/supination and wrist flexion/extension. Training is done bilaterally in a passive or active training mode. No feedback is given to the patient. BI-MANU-TRACK has been validated for subacute and chronic stroke patients in two CTs [149,126] and one RCT [127]. In total 66 persons after stroke were tested.

**BATRAC **[65] is an apparatus comprising of 2 independent T-bar handles that can be moved by the patient's hands (through shoulder and elbow flexion/extension) on a horizontal plane. Repetitive bilateral arm training is supported by rhythmic cueing and, where necessary, by assistance of movement. No patient feedback is provided. BATRAC has been tested for chronic stroke patients in one CT [65] and one RCT [67]. In total 37 patients were involved.

**ARMin **[150-153] is a semi-exoskeleton for movement in shoulder (3DOF), elbow (1DOF), forearm (1DOF) and wrist (1DOF). Position, force and torque sensors deliver patient-cooperative arm therapy supporting the patient when his/her abilities to move are inadequate. The combination of a haptic system with an audiovisual display is used to present the movement task to the patient. One small-scale CT [154] tested the clinical outcome of arm hand function in 3 chronic stroke patients after training with ARMin.

**NeReBot **[155,156] is a 3-degree of freedom robot, comprising of an easy to transport aluminum frame and motor controlled nylon wires. The end of each wire is linked to the patient's arm by means of a rigid orthosis, supporting the forearm. The desired movement is first stored into the system, by moving the patient's arm in a "learning phase" mode. Visual feedback comprises of graphical interface providing a 3D-image of a virtual upper limb on which 3 arrows show desired movement direction during movement. Auditory feedback accompanies the start and end of the exercise. NeReBot has been clinically tested in a RCT [156] involving 35 acute stroke patients.

**AJB or Active Joint Brace **[157] is a light-weight exoskeletal robotic brace that is controlled by means of surface EMG from affected elbow flexor and extensor muscles. It allows for assistance of movement in the elbow joint (1DOF). No feedback about exercise performance is provided. AJB has been tested in a small clinical study, involving 6 chronic stroke patients [157].

**T-WREX **is based on **Java Therapy**, that was developed by Reinkensmeyer et al [133]. T-WREX can train increased range of movement and more degrees of freedom, allowing for more functional exercising than Java Therapy does [19]. An additional orthosis can be used to assist in arm movement across a large, although not fully functional, workspace, with elastic bands to counterbalance arm weight. This makes it suitable for usage by patients with low muscle strength. Position sensors and grip sensors allow feedback on movement [133] and grip force [19]. T-Wrex aims to offer training of e.g. following activities: shopping, washing the stove, cracking eggs, washing the arm, eating, making lemonade. Limitations in movement of the shoulder (especially rotations) and forearm (no pro- or supination) cause a discrepancy between functional relevance of the exercise that is instructed and the actual movement that is performed.

Patients and therapists are presented with three types of progress charts: 1) frequency of system usage; 2) performed activity in comparison with customisable target score, average past performance and previous score; and 3) progress overview, which displays a graphical history of the user's scores on a particular activity [19,130,133]. T-Wrex has been validated through a clinical trial, involving 9 chronic stroke patients [19].

**UniTherapy **[158,159] is a computer-assisted neurorehabilitation tool for teleassessment and telerehabilitation of the upper extremity function in stroke patients. It makes use of a force-feedback joystick, a modified joystick therapy platform (TheraJoy) and a force-feedback steering wheel (TheraDrive).

Four operational modes are used: assessment mode; passive training mode; interactive mode (interaction with telepractitioner) and bi-manual mode (use of two force devices simultaneously).

UniTherapy provides visual and auditive cues in response to success/failure.

Although very engaging, UniTherapy offers movement therapy that is not task-oriented. Apart from moving a car steering wheel, as practised in TheraDrive (Driver's SEAT) [160,161], one can question transfer to skilled performance that is needed in everyday life. UniTherapy has been validated for chronic stroke patients in one CT [161] and one CCT [14], involving a total of 23 patients.

**Haptic Master **[144] is a three degrees of freedom robot, equipped with force and position sensors, that has been used for training arm movements of stroke patients [162-164]. A robotic wrist joint that provides one additional active and two passive degrees of freedom can extend it. All exercises happen in a virtual environment. Performance feedback is provided. The therapist can create virtual tasks. Three different therapy modes are implemented: the Patient Passive mode, the Patient Active Assisted mode and the Patient Active Mode. Therapy is, amongst others, focussing on task-oriented training in a 3D virtual environment as in the GENTLE/S project (reaching to a supermarket shelf, pouring a drink) [164] or focussing on task-oriented training with real object manipulation as done with ADLER (Activity of Daily Living Exercise Robot)[163]. A limiting factor for task-oriented training is the device's small range of motion. Two clinical trials provide evidence for improvement of arm hand function after use of haptic master training in subacute and chronic stroke patients [162,164]. In total 46 patients have been tested.

**Assisted Rehabilitation and Measurement Guide (ArmGuide) **is a 4 degrees of freedom robotic device, developed by Kahn et al. [165-168] to provide arm reaching therapy for patients with chronic hemiparesis. An actuator controls the position of the subject's arm, which is coupled to the device through a handpiece. This handpiece slides along a linear track in the reaching direction. Real time visual feedback of the location of the arm (along the track, elevation angles of track, target location) is given to the patient. ArmGuide has been tested in three clinical studies, involving in total 41 chronic stroke patients [165,167,169].

Virtual reality-based hand training systems that have been developed by Burdea et al. are **Rutgers Master II glove and Cyber Glove **[170,15,171]. Patients practise by doing one to four hand exercise programs in form of computer games. Each program focuses on different aspects of hand movement: range of movement, speed of movement, individual finger movement or finger strengthening. The exercises are aiming to have a task-oriented component (e.g. grasp virtual ball, piano) but are mostly analytic. Patients receive concurrent haptic feedback, visual feedback and auditory feedback on exercise performance. Also feedback about speed, range, and strength are provided real-time. In total, seven patients were included in two small-scale clinical trials [15,171].

#### Sensor-based training systems

Bonato [172] addressed the importance of developing wearable miniature monitoring devices, facilitating functional movement assessment in natural settings in an unobtrusive way. To date, although in full development (e.g. [173-175]), no such systems exist that have been clinically validated.

AutoCITE is a device that has been developed to automate constrained induced movement therapy [176,177]. It consists of a computer, a chair and 8 task devices (for reaching, tracing, peg board use, supination/pronation, threading, arc-and-fingers, finger tapping, and object flipping) that are organised on 4 work surfaces and are contained in a cabinet. The patient is guided through exercise instructions by the computer monitor. Performance variables are measured through built in sensors [56]. Video-conferencing equipment provides the patient with exercise instruction and bidirectional audio communication between therapist and participant. The patient receives prescriptive and descriptive, concurrent and terminal feedback of performance. Also reinforcing or encouraging feedback is given to address the motivational component of the training. The tool does allow for training at home by the patient, although some (remote) therapist supervision is still needed during training [176,177]. Thirty-four patients are involved in total in one controlled clinical trial and one clinical trial.

## Discussion: does technology use current insights in state-of-the-art approaches for motor (re)learning?

There has been a large evolution in rehabilitation technology in the last decade that has created a vast spectrum of new opportunities for patients and therapists. In order to evaluate this progress, strengths and weaknesses of current technology are assessed for each of the criteria that were presented in this paper (see table 1).

### Criteria relating to therapy aspects

#### Addressing function, activity and participation level

Most of rehabilitation technology has been developed based on existing (physical) interaction modes between therapist and patient [132]. Although task-oriented approaches are accepted as beneficial by persons who are involved in development of robotics [153,163] and are mentioned as a wishful trend for future technology development [97], most rehabilitation systems support analytical training methods (function level). To date, only T-WREX, ADLER, TheraDrive, ARMin and AutoCITE aim to offer task-oriented training for the upper extremity. Reviewing the results of clinical trials on training with robotics, substantial improvements in short-term and long-term strength and analytical upper limb movements have been shown in stroke patients. However, while waiting for more clinical trial results of robotics that include task oriented training, experimental evidence indicates that to date robotic upper limb training fails to transfer to improvement of the activity level [137,178,19]. From evidence obtained via functional neuroimaging it is known that functional recovery from stroke is positively influenced by task-specific sensorimotor input through training [72] or everyday use [73,74]of the arm and hand. It seems that the impact of rehabilitation technology on functional outcome could be optimised by offering more chances to the nervous system to experience "real" activity-related sensorimotor input during training of upper limb movement.

Nevertheless, the state-of-the-art robotic upper extremity training in stroke patients can play a very important role in alleviating therapists from administering repetitive analytical exercises and can be useful in combination with other conventional treatment [156]. Hesse [179] advocates robotics as an ideal means of training for severely affected patients where external assistance such as actuator assistance to movement and/or exoskeleton support may overcome problems of muscle weakness. Mildly affected patients do not need such assistance and benefit more from task-oriented training approaches.

Most robotic systems to date focus on the proximal part of the upper limb (MIME, T-Wrex, and ArmGuide). Rutgers Master II is focussing exclusively on hand and fingers.

Of the current robotic systems, only ADLER allows for training of the entire arm (shoulder, elbow, forearm, wrist) and hand; which means that for most robotic systems it is difficult to train meaningful upper extremity skills as they occur in every day life [132,178]. The MIT-MANUS team is developing a hand module to complete the existing upper limb robot [125]. MIT-MANUS will be allowing training of the upper extremity over all its joints, albeit not possible to train all joints of the upper extremity at the same time. This implies that training a skill is only possible in some of its broken down components.

Also training in full range of joint motion and with all necessary degrees of freedom is not possible with any of the existing robotic systems; which is again a limiting factor of current robotic systems to allow for task-oriented training.

Different robotic systems train different body areas, with different kind of exercises and feedback. Therefore, the concept of Krebs [125] to have a "gym" or exercise room in which patients can use several kinds of robotics to train, or the concept of Johnson [159] to have an "integrated suite of low-cost robotic/computer assistive technologies" is a good approach. This kind of training does practise very essential components of movement, such as muscle strength and range of movement and can be very useful in support of training in a rehabilitation setting. However, this solution is still not offering training of movement strategies that enable learning of skilled arm-hand performance, as is the purpose of task-oriented training. For practical (e.g. patient independence for use) and cost reasons it is also unlikely to become a solution for the home environment.

Sensor-based solutions have potential to offer treatment that may influence impairment, activity and participation level. These possibilities have though not been fully used so far. AutoCITE does provide skill training, albeit to date for a limited number of skills (threading, tracing, reaching, object flipping, displacement of pegs), and has proven to influence activity level [177].

#### Offering environmentally contextual training

Kahn et al [41] found better outcome effects after training chronic stroke patients for reaching movements without use of robotics than for patients who actually practised with robotics. These findings promote systems that allow training of skills in their natural environment. In this sense, sensor-based-solutions can potentially support environmentally contextual training more than robotics do. The robotic system that allows most for environmentally contextual training is ADLER [163], as the hand is left free to allow for object manipulation. This feature is missing in, e.g. T-WREX [19]where forces are applied on a handgrip. To provide realistic sensorimotor input and encourage task-related problem solving, robotic systems research may benefit from the use of mixed reality systems (e.g. concept of Edmans et al [180]), where movement sensitive objects and machine vision allow for a virtual reality environment that is steered by "real" object manipulation.

The sensor system AutoCITE allows for object manipulation, although is limited to chair seated training in front of work surfaces in a cabinet, which may hinder transfer effects to "everyday situations". On the other hand, the progress of seven chronic stroke patients on Motor Activity Log testing after training with AutoCITE does suggest positive effects on everyday life use and usefulness of the affected limb [177].

#### Inclusion of frequent movement repetition

Robotics are very suitable for facilitating repetitive training in stroke patients with all functional levels [156], which has proven to address brain plasticity and to improve function [9]. For sensor-based solutions, only stroke patients who have a certain level of endurance and muscle strength (should be able to move against gravity) can be instructed to repeat a movement frequently.

#### Patient and goal-tailored training load & Exercise Variability

Most robotic systems (especially MIT-Manus, Haptic Master and MIME) are very suitable for delivering a patient-tailored and goal-tailored training load. Actuators can deliver assistance for movement execution where necessary and resistance where possible. This makes robotic systems very valuable for arm and hand function training of patients with lower functional levels. Fine-tuned assistance encourages patients to use all their capabilities to progress movement performance. Such strong feature is absent in sensor-based solutions.

As for training variability, robotics do provide a large variability for analytical exercises. Exercise variability is currently especially limited for stroke patients with higher functional levels, who need more challenge. Also sensor-based solutions, although having a large potential for variability of patient-tailored functional exercises, seem not to have been able to date to actually offer this to patients yet.

### Criteria related to motivational aspects

#### Gaming

All robotic systems described in this paper include gaming aspects in their upper limb rehabilitation for stroke patients. Current sensor-based training systems are (still) focussing mostly on instruction of analytical movement.

#### Therapist independence

Most of the current technological solutions still need therapist help to attach the technology to the patient, and/or to operate the technology. In practice this means that these technologies can be useful in rehabilitation centres allowing a therapist to supervise several patients at the same time. But as the duration of hospitalisation or stay at rehabilitation centre becomes compressed, patients are increasingly left "home alone".

#### Active role of the patient in rehabilitation

There is strong evidence that specific and difficult goals can improve patient performance [84]. Patient customisation of treatment refers to exercises that are meaningful to the patient [181] and to a training load that is tailored to patient capabilities (percentage of repetition maximum (RM) of the patient [79,182]) as well as to treatment goals (increase of muscle force, endurance, coordination) [79]. Technology should be able to offer exercises that are close to what the patient prefers to train on [181]. Few applications offer enough exercise variability to support individual goal setting according to individual needs. From the description in the related articles, it cannot be understood which training load (e.g. maximum load that a patient can perform a certain amount of times before needing a rest) [79,182]) has been applied and how this has been customised to the patient.

Even when the treatment on offer is patient-customised, the principles that exercise programs are based on should be generic, allowing for inter-individual comparison. Examples of such principles are: a) the method for setting treatment goals (e.g. goal attainment scaling [88]), b) exercise programs that are designed in function of certain treatment goals [79], and c) the use of uniform and appropriate assessment tools [23,183,184]. When these are taken into account, treatment can be evaluated to give adequate patient feedback on individual progress, as well as allowing for clinical research into the effect of customised treatment methods, whether they are technology-supported or not.

### Criteria related to feedback on exercise performance

Most technological applications provide good assessment of exercise performance; allowing for objective and valid feedback. It is not always clear from the description in articles how this assessment of performance is used in order to give feedback. Another problem to be identified here is, that most assessment is done at the function level only (UniTherapy, MIT Manus, MIME) and can therefore only be used to limited extent as feedback for skill training. Most systems provide the patient with feedback; either during exercise performance (MIT Manus) or terminal (T-WREX, UniTherapy) or both (AUTOCITE, Rutgers Master II & CyberGlove).

## Conclusion

In the light of the fast developments in rehabilitation technology, it is useful to reflect on guidelines that allow future technologies to offer engaging rehabilitation with optimal training possibilities. This review confirms the commentary of Johnson [97] that technology for supporting upper limb training after stroke needs to align with the evolution in the field of rehabilitation towards functionally oriented approaches that influence function level, activity level and participation level. The review offers an inventory of points to focus on for development of future and/or adaptation of current rehabilitation technology.

Motor learning may be further improved when feedback progress criteria could be fitted to certain patient types, depending on type and lesion location and to the different phases of motor learning (e.g. as described by Fitts and Possner [92]), thus facilitating feedback delivery most appropriate for the patient.

According to the present literature, it is not yet understood how different rehabilitation approaches contribute to restorative processes of the central nervous system after stroke. A contributing factor to the success of task-oriented approaches may be found in the task-specific sensorimotor input that shapes brain reorganisation in such a way that it can be supporting restitution or substitution of skilled arm hand function. Research, as currently ongoing in, e.g., the EXPLICIT-stroke trials [185-187], will shed more light on training related neurologic changes that are responsible for the improvement of function and activity after stroke.

Although a number of rehabilitation technology approaches show promising results in small-scale studies, it will be interesting to have results from large scale clinical trials. It is advocated that future trials include outcome assessment of arm-hand function on all ICF-levels [23,183,184] to give evidence for the influence of technology-supported training on skilled arm-hand function and patient participation, as well as on function level. Future trials should also report the patients' goals that are trained and the individual patient training load and exercise programs that are used in order to allow for comparison between different studies.

Finally it must be mentioned that rehabilitation technology that has not been clinically reported until 2007 and therefore was not reviewed in this study, represents a lot of potential for rehabilitation in the future.

## Competing interests

The authors declare that they have no competing interests.

## Authors' contributions

AAT and HAS have made substantial contributions to conception, design and drafting the manuscript. All authors have been involved in critically revising for important intellectual content. HAS and HK have given the final approval for publication.

## Supplementary Material

Additional file 1 **Overview of upper extremity rehabilitation robotics for stroke patients that have been tested through 1 or more clinical trials**. This file gives an overview of all robotic systems that have been tested through clinical trials, controlled clinical trials or randomized controlled clinical trials between 1997 and 2007. Click here for file
