# Supplementary material for: Technology-assisted training of arm-hand skills in stroke: concepts on reacquisition of motor control and therapist guidelines for rehabilitation technology design
Source: J Neuroeng Rehabil. 2009 Jan 20;6:1. doi: 10.1186/1743-0003-6-1 (PMC2647548; doi:10.1186/1743-0003-6-1)
Supplement: Additional file 1 — Overview of upper extremity rehabilitation robotics for stroke patients that have been tested through 1 or more clinical trials. This file gives an overview of all robotic systems that have been tested through clinical trials, controlled clinical trials or randomized controlled clinical trials between 1997 and 2007. [file 1743-0003-6-1-S1.pdf]

Additional file 1 - Overview of upper extremity rehabilitation robotics for stroke patients that have been tested through one or more clinical trials

(FB= feedback, DF= Degrees of freedom, PA= Physiotherapy Approach, A= Analytical, T= Task-oriented, TDL= therapist dependency level: 0=no, 1=minimal 2=fully dependent, OCM= outcome measure, CT= clinical trial, CCT= controlled clinical trial, RCT= randomized controlled clinical trial, AS=Ashworth Scale, FM=Fugl Meyer Assessment, FIM= Functional Independence Measure, MRC=Medical Research Council motor power score, MSS=Motor Status Scale, MP=Motor Power Scale, AMAT=Arm Motor Ability Test, WMFT=Wolf Motor Function Test, SIS=Stroke Impact Scale, RMA= Rivermead Motor Assessment, TCT= Trunk Control Test, RFT= Rancho Functional Test, CMM= Chedoke-McMaster test, EMG= electromyography, BBT = Box and Blocks test, mBBT= modified Box and Blocks test, JT= Jebson Test, UMAQS= University of Maryland Arm Questionnaire, KM= kinematic information).

Name (total amount of patients tested)	Body area trained	Modalities	DF	P-A	FB	TDL	CT CCT RCT (n patients)	OCM	Acute subacute chronic patients
**MIT-Manus (372)**	shoulder elbow wrist (hand)	passive active interactive: movement- or emg-triggered	2	A	Concurrent: visual tactile auditory	2	CT (30) [135]	FM,FIM, MP,MSS	chronic
							CT (30) [136]	MSS,FM, WMFT	chronic
							CT (15) [137]	FM,MP, WMFT, SIS,KM	chronic
							CT(3) [131]	EMG	subacute
							CT(117) [138]	FM, KM	chronic
							RCT(20) [139]	FM,FIM	acute
							RCT (56)[140]	FM,FIM MSS, MPS	acute
							RCT(12)[141]	AMAT FM, KM	chronic
							RCT(42)[142]	AS,MRC FM,MSS	chronic
							RCT(47) [143]	FM,KM	chronic
**MIME (76)**	shoulder elbow forearm	passive active-assisted active-constrained bimanual	6	A	none	2	CT (13) [147]	KM	chronic
							RCT (21)[146]	FM	chronic
							RCT (27)[145]	FM FIM	chronic
							RCT (15)[148]	FM FIM	subacute
**BI-MANU-TRACK (66)**	forearm wrist	bimanual: passive or active	1	A	none	1	CT (10) [149]	FM	subacute
							CT(12) [126]	MAS, RMA	chronic
							RCT(44)[127]	FM, AS MRC	subacute
**BATRAC (37)**	shoulder elbow	active active assisted	1	A	none	1	CT (16) [65]	FM, WMFT UMAQS	chronic

							RCT (21) [67]	FM,<br>WMFT<br>UMAQS<br>fMRI<br>EMG	chronic
**ARM-in<br>(3)**	shoulder<br>elbow<br>forearm<br>wrist	active<br>passive<br>interactive	6	T<br>A	position<br>force	2	CT (3) [154]	FM<br>KM	chronic
**NeReBot<br>(35)**	shoulder<br>elbow	active<br>active assisted<br>passive	3	A	visual<br>auditory<br>KP	2	RCT (35)[156]	FM MRC<br>FIM<br>TCT<br>MAS	acute
**AJB<br>(6)**	Elbow	active<br>active assisted	1	A	-	1	CT (6) [157]	FM<br>MAS	chronic
**T-Wrex<br>(9)**	shoulder<br>elbow<br>forearm<br>wrist	passive<br>interactive:<br>active, active-assisted,<br>resisted	2	T<br>A	position<br>grip force<br>coordination<br>speed	1-2	CT (9) [19]	FM, BBT,<br>MBBT,<br>RFT	chronic
**UniTherapy<br>(23)**	shoulder<br>elbow<br>forearm	passive<br>interactive:<br>active, active-assisted,<br>resisted	2	A	terminal FB:<br>visual<br>auditory		CCT (16) [14]	FM,<br>KM,<br>EMG	chronic
							CT (7) [161]	FM,<br>KM	chronic
**Haptic<br>Master<br>(46)**	shoulder<br>elbow<br>forearm	passive<br>interactive:<br>active; active-assisted,<br>resisted	3<br>+3	T<br>A	_	2	CT (31) [164]	FM, MAS	chronic
							CT (15)[162]	MRC,<br>KM	subacute
**ArmGuide<br>(41)**	shoulder	passive<br>active, active-assisted<br>,resisted	4	A	concurrent:<br>visual	2	CT (1) [167]	KM	?
							CT (19) [168]	RFT,<br>CMM,<br>KM	chronic
							RCT (21)[169]	FM,<br>WMFT,<br>RLA	chronic
**RM II<br>(7)**	hand	interactive:<br>active<br>active-resisted		A<br>T	terminal KR &<br>KP<br>concurrent:<br>force,auditory<br>visual	0	CT (4) [171]	KM, JT	chronic
							CT (3) [15]	KM, JT	chronic
